# Treatment outcomes of pathological fractures in patients with benign bone tumors

**DOI:** 10.1097/MD.0000000000041584

**Published:** 2025-02-14

**Authors:** Kazuhiko Hashimoto, Shunji Nishimura, Tomohiko Ito, Ryosuke Kakinoki, Koji Goto

**Affiliations:** a Department of Orthopedic Surgery, Kindai University Hospital, Osaka-Sayama, Osaka 589-8511, Japan.

**Keywords:** benign, bone tumor, impending fractures, pathological fractures, surgical treatment

## Abstract

Pathological fractures of benign bone tumors can be difficult to treat, and the underlying pathogenesis remains unclear. Herein, we aimed to determine preventive measures for pathological fractures in patients with benign bone tumors based on fracture outcomes. Between April 2015 and July 2023, we enrolled 18 consecutive patients with oncological pathological fractures treated at our department. Age, sex, histopathological diagnosis, site of origin, whether incisional or pathological fracture, treatment, operative time, blood loss, recurrence, and characteristics of impending and pathological fractures were examined. The median patient age was 22 years, comprising 9 males and 9 females. The pathology included bone cysts (n = 6), enchondromas (n = 5), fibrous dysplasia (n = 4), giant cell tumors (n = 2), and aneurysm bone cysts (n = 1). Six cases involved the humerus, 5 the femur, 3 the phalanges, 2 the toes, 1 the ribs, and 1 the tibia. Five and 13 cases were impending and pathological fractures, respectively. Thirteen patients underwent surgery, whereas 5 were treated conservatively. Surgical methods included curettage and artificial bone graft (n = 6); curettage and artificial bone graft plus compression hip screw fixation (n = 3); and curettage and artificial bone graft plus plate fixation, intramedullary nail, artificial head replacement, and plate fixation (n = 1 case each). The mean operative time and blood loss were 76 ± 56 minutes and 10 ± 80.1 mL, respectively. Recurrence occurred in 1 case. All impending fractures had onset in the lower extremity bones. Pathological fractures due to benign bone tumors of the lower extremities should not be overlooked as symptoms of pain.

## 1. Introduction

Pathological fractures may arise from a variety of conditions, ranging from bone tumors to metabolic diseases and infections.^[1]^ Additionally, pathological fractures may occur irrespective of whether the underlying bone tumors are benign or malignant. Fractures due to both benign and malignant bone tumors should be identified and managed appropriately by the attending orthopedic surgeon.^[1,2]^ The most common benign bone tumors resulting in pathological fractures include bone cysts, aneurysmal bone cysts (ABCs), non-ossifying fibromas, and fibrous dysplasia (FD),^[2]^ which should be promptly recognized and treated. In most benign cases, the fractures heal, and lesions can be addressed at the time of the fracture or after the fracture has healed.^[1]^ A thorough history, physical examination, and simple radiographic analysis are important to identify the underlying cause and guide treatment.^[1,2]^ However, detailed literature on pathological fractures remains insufficient. In the current study, we aimed to provide a detailed report on pathological fractures treated in our department.

## 2. Materials and methods

### 2.1. Subjects

Between April 2015 and July 2023, 18 consecutive patients with oncological pathological fractures were enrolled in this study. Age, sex, histopathological diagnosis, site of origin, whether the fracture was incisional or pathological, treatment, operative time, blood loss, and recurrence were investigated. We also examined the characteristics of impending and pathological fractures. All patients treated during the study period were included. Patients in whom the course of treatment could not be followed were excluded. Patients whose clinical course could not be adequately monitored or who did not undergo appropriate imaging tests were withdrawn from the study. This ensures that only participants with complete and reliable data are included in the final analysis, maintaining the integrity of the research outcomes. Ethical approval for this study was obtained from the Ethics Committee of Kindai University Hospital (approval no: 31-153; Osaka, Japan).

### 2.2. Statistical analyses

The χ-square test was used to compare the number of cases of pathological and incisional fractures between tumors in lower extremity bones and those in other bones.

Data analyses were performed using Stat Mate 5.05 (ATMS, Tokyo, Japan).^[3]^

## 3. Results

Table 1 summarizes the characteristics of the patient population. The median patient age was 22 years (range, 11–77 years). Nine patients were male, and 9 were female. The pathology included bone cysts in 6 cases, enchondroma in 5 cases, FD in 4 cases, giant cell tumor (GCT) in 2 cases, and aneurysm bone cyst in 1 case. Six cases involved the humerus, 5 the femur, 3 the phalanges, 2 the toes, one the ribs, and 1 the tibia. Five and 13 cases were impending and pathological fractures, respectively. A sum of 13 patients underwent surgery, and 5 were treated conservatively. The surgical methods included curettage and artificial bone graft in 6 cases; curettage and artificial bone graft plus compression hip screw fixation in 3 cases; and curettage and artificial bone graft plus plate fixation, intramedullary nail, artificial head replacement, and plate fixation in 1 case each. The mean operative time was 76 ± 56 minutes (mean ± standard deviation), and the mean blood loss was 10 ± 80.1 mL. 1 case of recurrence was observed. Considering bone tumors of the lower extremity, 3 cases of pathological fractures and 5 cases of impending fractures were recorded. In bone tumors other than those of the lower extremities, there were 10 cases of pathological fractures and none of impending fractures. Bone tumors of lower extremity bones were associated with significantly more impending fractures than bone tumors in nonlower extremity bones (χ-square test: *P* = .008). All patients with impending fractures had onset in the lower extremity bones.

**Table 1:** Characteristics of the study population.

Factor	Patients, n
Age (mean, yr)	22
≤20	7
>20	11
Sex	
Males	9
Females	9
Fracture site	
Upper limb	9
Lower limb	8
Trunk	1
Type of histology	
Bone cyst	6
Enchondroma	5
FD	4
GCT	2
ABC	1
Operation (mean ± standard deviation)	
Time (min)	76 ± 56
Blood loss (mL)	10 ± 80.1
Recurrence	
+	1
−	17

ABC = aneurysmal bone cyst, FD = fibrous dysplasia, GCT = giant cell tumor.

## 4. Discussion

Pathological fractures of benign bone tumors are often difficult to treat, and detailed mechanisms underlying their pathogenesis remain unclear. Herein, we reviewed the details of cases of pathological fracture in patients with benign bone tumors treated at our institution.

Between the ages of 5 and 10 years, the most common pathologies include unicameral bone cysts, ABCs, non-ossifying fibromas, osteochondromas, and enchondromas. Unicameral bone cysts, ABCs, non-ossifying fibromas, osteochondromas, FD, chondroblastomas, and GCTs are common in patients aged 10 to 20 years.^[1,2]^ Non-ossifying fibromas, also known as fibrous cortical defects or non-osteoplastic fibromas, are the most common benign bone tumors in children, estimated to occur in 30% to 40% of all cases.^[1]^ The prevalence of primary ABCs is approximately 2% of all benign bone tumors.^[1]^ Eighty percent of cases are diagnosed in patients under 20 years of age.^[4]^ FD, a benign nonhereditary disease, destroys fibrous tissue and small normal bones and has a prevalence of 5% to 7% among benign bone tumors.^[5]^ Enchondromas are benign cartilaginous tumors occurring within the bony medulla, constituting approximately 90% of benign tumors in the hand.^[6]^ GCTs of the bone account for approximately 15% to 20% of all benign bone tumors and approximately 4% to 5% of all bone tumors.^[7]^ The present study included a similarly large number of bone cysts and endochondromas, although the cases were relatively old.

Pathological fractures reportedly occur in approximately 50% of patients with solitary bone tumors, particularly in the proximal femur.^[8]^ These fractures are particularly prevalent in the proximal third of the femur or metaphysis of the tibia. Lesions of the load-bearing bones of the lower extremities pose a substantial risk of fracture.^[1]^ In the current study, all impending fractures were found in the femur. Because the lower extremity bones are load-bearing bones, they were easily detected at the incisional fracture stage.

Two main treatment modalities exist: conservative and surgical.^[1,2]^ The first priority is treating the tumor and its extension. Fracture treatment is the second priority. Internal fixation of pathological fractures is prohibited because it contributes to the spread of tumor cells.^[9,10]^ However, when addressing unicameral bone cysts, the priority is to treat the fracture, followed by tackling the lesion site.^[1]^ Fracture treatment is simple, that is, immobilization of the extremity for 4 to 6 weeks^[1]^; this applies to fractures in non-weight-bearing areas. Patients with cysts in the proximal femur are more prone to pathological fractures; however, they are also more likely to experience fractures and heal when compared with patients with cysts in the proximal humerus.^[11]^ Most fractures heal, but unicameral bone cysts persist in 20% to 50% of cases.^[1]^ The ultimate goal of treatment is also to eradicate and prevent cyst formation. Current treatment options to address unicameral bone cysts include some combination of the following^[1]^: decompression or mechanical destruction of the cyst wall, injections (steroids, bone marrow puncture, demineralized bone matrix, or bone substitute), and structural support in weight-bearing bones for immobilization.^[1]^ Surgery should be the treatment of choice in the case of an unstable fracture or involvement of a weight-bearing bone.^[1]^ Treating the cyst in parallel with or after fixation would be advisable. In the present study, one patient was treated conservatively based on the patient’s refusal to undergo surgery. In other cases, internal or external fixation was performed. Fracture treatment is a priority in the management of endochondromas, with secondary deformities treated subsequently.^[2]^ Typically, curettage and bone grafting are performed,^[12,13]^ and the same treatments were employed in the current study. ABCs lead to pathological fractures in approximately 36% of patients,^[1]^ and pathological fractures due to ABCs are often treated simultaneously with the lesion, given that the lesion does not heal with fracture healing.^[1,7]^ Curettage and bone grafting of the lesion are the most widely accepted management strategies, and patients in the current study received similar treatment.^[14]^ Surgical treatment of FD essentially requires curettage and internal fixation of the lesion.^[1]^ External fixation with a plate or compression hip screw may be necessary, depending on the fracture site.^[1]^ However, in moderate to severe FD, fusion or fracture under the plate can lead to Shepherd’s crop deformities.^[1,8,15]^ In FD cases included in the current study, fixation was performed in 2 of the 4 cases, and 1 case was treated conservatively owing to involvement of the torso. One femur case exhibited a severe deformity and was treated conservatively with immobilization instead of fixation. Treatment of pathological fractures associated with GCTs may involve denosumab administration, in addition to lesion curettage and bone grafting.^[16,17]^ In the present study, no patient had received denosumab therapy. One case, which extended from the femoral neck to the metaphyseal region, was treated with artificial head replacement to ensure complete lesion removal.

The recurrence rate/persistence of bone cysts ranges from 6% to 30%, depending on the surgery.^[1,18,19]^ A 94% cure rate has been reported after the initial surgery, and a 100% cure rate can be achieved after 2 surgeries.^[19]^ Omlor et al^[20]^ reported 1 recurrence among 42 cases with enchondroma resection, bone grafting, and plate fixation. Errani et al^[21]^ documented a recurrence rate of 11%. ABCs have been associated with a recurrence rate of approximately 20%.^[7,22]^ Osaka E et al^[16]^ found that 18 of 27 patients (66.6%) with FD and Shepherd’s crook deformities, who were treated surgically using several different methods, required repeat surgery or casting owing to recurrence or microfractures. Conversely, Yang et al^[23]^ reported no progression in all 14 patients treated surgically using the 4-step procedure for the lesion, valgus osteotomy for correction of the deformity, and intramedullary nail with neck cross pinning. FD resulting in Shepherd’s deformities may be difficult to treat. Gillani et al^[24]^ reported recurrence in 3 of 40 patients after resection and bone cementation of GCTs. Van der Heijden et al^[25]^ documented a recurrence rate of approximately 30%. Importantly, whether denosumab can reduce the relapse rate remains controversial.^[25]^

This study has some limitations. First, the sample size was small. Second, this was a retrospective study. Third, no comparisons with nonfractured cases were performed. These limitations collectively impact the study’s technical validity, robustness of supporting data, and overall experimental rigor, potentially affecting the strength and applicability of the conclusions drawn. To address these shortcomings and enhance the reliability of the results, we recommend conducting a prospective study with a larger, more diverse sample size. This future research should include a comparison between pathological and non-pathological fractures, thereby providing more comprehensive and generalizable insights into the subject matter.

Pathological fractures in benign bone tumors may be preventable, especially in the lower extremity bones. With a reliable diagnosis, benign pathological fractures should be detected at the impending fracture stage. Once diagnosed, appropriate treatment should be administered according to the histopathological characteristics of the individual.

## Acknowledgments

The authors would like to thank Editage (www.editage.jp) for the English language editing.

## Author contributions

**Conceptualization:** Kazuhiko Hashimoto, Shunji Nishimura, Tomohiko Ito, Ryosuke Kakinoki, Koji Goto.

**Data curation:** Kazuhiko Hashimoto, Shunji Nishimura, Tomohiko Ito, Ryosuke Kakinoki, Koji Goto.

**Formal analysis:** Kazuhiko Hashimoto, Tomohiko Ito.

**Investigation:** Kazuhiko Hashimoto, Shunji Nishimura, Tomohiko Ito, Ryosuke Kakinoki, Koji Goto.

**Methodology:** Kazuhiko Hashimoto, Tomohiko Ito, Ryosuke Kakinoki.

**Project administration:** Kazuhiko Hashimoto, Shunji Nishimura, Tomohiko Ito, Ryosuke Kakinoki, Koji Goto.

**Resources:** Shunji Nishimura.

**Software:** Kazuhiko Hashimoto.

**Supervision:** Kazuhiko Hashimoto, Shunji Nishimura, Tomohiko Ito, Ryosuke Kakinoki, Koji Goto.

**Validation:** Kazuhiko Hashimoto, Ryosuke Kakinoki, Koji Goto.

**Visualization:** Kazuhiko Hashimoto, Shunji Nishimura, Tomohiko Ito, Ryosuke Kakinoki.

**Writing – original draft:** Kazuhiko Hashimoto, Shunji Nishimura, Tomohiko Ito, Ryosuke Kakinoki, Koji Goto.

**Writing – review & editing:** Kazuhiko Hashimoto, Shunji Nishimura, Tomohiko Ito, Ryosuke Kakinoki, Koji Goto.
